# Immunogenicity and safety of concurrent or sequential administration of live, attenuated SA 14-14-2 Japanese encephalitis vaccine (CD-JEV) and measles-mumps-rubella vaccine in infants 9–12 months of age in the Philippines: A non-inferiority Phase 4 randomized clinical trial

**DOI:** 10.1016/j.jvacx.2020.100074

**Published:** 2020-08-14

**Authors:** Maria Rosario Capeding, Edison Alberto, Jodi Feser, Jessica Mooney, Yuxiao Tang, Susette A. Audet, Judy A. Beeler, Damon W. Ellison, Lei Zhang, G. William Letson, Kathleen M. Neuzil, Anthony A. Marfin

**Affiliations:** a Research Institute for Tropical Medicine, 9002 Research Drive, Filinvest Corporate City, Alabang, Muntinlupa City, Manila 1781, Philippines; b PATH, PO Box 900922, Seattle, WA 98109, USA; c US Food and Drug Administration, 10903 New Hampshire Avenue, Silver Spring, MD 20993, USA; d Armed Forces Research Institute of Medical Sciences, 315/6 Rajvithi Road, Bangkok 10400, Thailand; e Chengdu Institute of Biological Products Co., Ltd., 379, 3rd, Jinhua Road, Jinjiang District, Chengdu 61002, China

## Abstract

**Introduction:**

Japanese encephalitis (JE) virus is the leading cause of viral encephalitis across temperate and tropical zones of Asia. The live attenuated SA 14-14-2 JE vaccine (CD-JEV) is one of three vaccines prequalified by the World Health Organization (WHO) to prevent JE. When incorporating a new vaccine into a country’s Expanded Program on Immunization (EPI), it is important to show that the new vaccine can be administered concurrently with other routine pediatric vaccines without impairing the immune responses or changing the safety profiles of the co-administered vaccines. This Phase 4 open-label study evaluated the safety and immunogenicity of measles-mumps-rubella (MMR) vaccine co-administered with CD-JEV.

**Methods:**

The study randomized 628 healthy Filipino children aged between 9 and 10 months to receive MMR and CD-JEV concurrently or separately. MMR immunogenicity was measured 56 days after MMR vaccination using a measles plaque reduction neutralization test (PRNT), anti-mumps immunoglobulin G (IgG) enzyme-linked immunosorbent assay (ELISA), and anti-rubella IgG ELISA, respectively. Neutralizing antibody against JE virus was measured 28 days after CD-JEV vaccination using PRNT. Safety was assessed through solicitation of immediate reactions, adverse events (AEs) within 14 days of vaccination, unsolicited AEs occurring within 28 days, and serious adverse events (SAEs) during participation in the study**.**

**Results/Conclusions:**

During the study, no post-vaccinal encephalitis cases or related SAEs were reported in either group. Concurrent immunization with CD-JEV and MMR vaccines was not associated with any unusual safety signals when compared with sequential immunization. No significant differences between the regimens were seen in seropositivity or serology titer/concentration results for any of the antigens. Co-administration of CD-JEV and MMR was non-inferior to single administration of either vaccine.

## Highlights

• Live, attenuated SA 14-14-2 JE vaccine is immunogenic when given with MMR vaccine.
• Live, attenuated SA 14-14-2 JE vaccine is safe when given with MMR vaccine.
• Live, attenuated SA 14-14-2 JE vaccine may be coadministered with MMR vaccine.

## 1 Introduction

Japanese encephalitis (JE) virus is a flavivirus that causes devastating neurological disease resulting in mortality rates of 20–30% and neurologic sequelae in 30–50% of survivors [1]. The severity and duration of sequelae, together with an estimated 69,000 cases per year in endemic Asian countries, makes JE an important vaccine-preventable disease [2], [3]. JE vaccine is given in many Asian countries when children are 8- to 12-months-old, the same age when measles-containing vaccines (MCVs) are typically given in Asia. An increasing number of JE-endemic countries have introduced or will introduce measles-mumps-rubella (MMR) vaccine as the MCV within the Expanded Program on Immunization (EPI) schedule [4], [5]. Measles vaccine was given to 9-month-old children in the Philippines at the time of this study. Since completion of this study, the Philippines has integrated MMR into the EPI schedule.

Globally, the most widely used JE vaccine is SA 14-14-2 (CD-JEV), a live attenuated JE vaccine manufactured by Chengdu Institute of Biological Products (CDIBP) in China and prequalified by World Health Organization (WHO) in 2013. Approximately 400 million doses were used domestically in China and internationally in the decade prior to prequalification [2]. Studies have supported co-administration of CD-JEV with MCVs [6], [7], [8], [9]. Two previous studies have shown non-inferiority of the immune responses when MCV and CD-JEV were co-administered compared to sequential vaccination [7], [8], [9]. While these studies suggest that there would be similar non-inferiority between co-administered CD-JEV and MMR, it is uncertain whether the mumps antigen might alter the outcome.

The primary objective of this study was to demonstrate non-inferiority of response to measles and rubella antigens when MMR is given concurrently with CD-JEV. The secondary objectives were to demonstrate non-inferiority of response to mumps antigen and JE antigen when co-administered compared to the response when administered separately, compare any difference in magnitude of the serologic response, and compare relative safety between the two groups.

## 2 Methods

### 2.1 Study design and population

This study was a Phase 4 open-label trial conducted from October 2016 through July 2017 in which 628 Filipino 9-month-old infants were randomized 1:1 to one of two arms. Group 1 subjects (314 children), received one dose of CD-JEV vaccine and one dose of MMR vaccine simultaneously in different limbs on Day 0. Group 2 subjects (314 children), received one dose of MMR vaccine on Day 0 and one dose of CD-JEV 56 days later. Both groups received a second MMR dose per the routine immunization schedule on Day 84 of the study when the infants were 12 months old. Laboratory technicians conducting immune response analyses were blinded to group assignment.

Enrollment criteria included being a healthy 9-month-old infant residing in catchment areas of the Bayanan or Putatan community health centers (Barangay) in Muntinlupa City, Philippines. Study procedures, data collection, and maintenance of records and specimens were carried out in the health centers and at the Research Institute for Tropical Medicine (RITM). Exclusion criteria included prior receipt of any MCV or JE vaccine; known natural infection with measles, mumps, rubella or JE viruses; known hypersensitivity to any study vaccine components; prior use of investigational drugs within 90 days; or use of immunoglobulin or blood products in the preceding 90 days or during the study.

On-site study staff used random permuted block design stratified by site with block sizes of 4, 6, and 8 and a masked group allocation log to assign subjects to their respective groups. Enrollment of 628 participants (314 per group) gave this study an overall 90% power to detect a non-inferiority margin of 10% with a one-sided type-one error rate <2.5%, assuming 95% and 90% seropositivity rates for measles and rubella vaccines when administrated alone, respectively, and approximate 20% non-evaluable rate inclusive of any lost to follow-up. Sample size calculations and confidence intervals were based on Farrington-Manning score test [10].

### 2.2 Vaccines and immunization procedures

CD-JEV was supplied in 5-dose vials as a lyophilized powder and separate diluent, lot numbers 201511C090-2 and 201510C77, respectively. Each 0.5 mL dose for subcutaneous injection contains not less than 5.4 log PFU of live JE virus. After reconstitution, study nurses administered a single 0.5 mL dose of CD-JEV to subjects by subcutaneous injection in the left upper thigh. The remaining doses in the 5-dose vial were then discarded.

MMR vaccine was supplied in its single-dose presentation as a lyophilized cake with diluent, lot number A69CE107A manufactured by GlaxoSmithKline, Inc. Each dose reconstituted in a volume of 0.5 mL contains not less than 1000 CCID50 of Schwartz measles virus, 5012 CCID50 of RIT 4385 mumps virus, and 1000 CCID50 of Wistar RA 27/3 rubella virus. After reconstitution, study nurses administered a single 0.5 mL dose of MMR vaccine to subjects by subcutaneous injection in the right upper thigh.

### 2.3 Immunogenicity assessment

Measles antibody is usually measured at 0 and 28 (+7) days; however, since rubella immunogenicity is best measured at least 8 weeks after immunization and since immunity to measles or mumps was not expected to wane between 28 and 84 days, blood was collected at 56 (+7) days post-vaccination to capture the rubella response at an optimal time [11], [12], [13]. JE antibody was measured at 0 and 28 (+7) days post-vaccination [14]. Measles antibody response was measured by WHO-standardized plaque reduction neutralization test (PRNT) with ND50 titers converted into concentration of measles antibody in international unitage relative to the performance of NIBSC 97/648 Reference Serum (Third International Standard) tested in parallel; rubella IgG antibody response was measured using an indirect enzyme-linked immunosorbent assay (ELISA) (ZEUS Rubella IgG ELISA, 9Z9801G; Branchburg, New Jersey); and the mumps IgG antibody response was measured using a qualitative ELISA (Mumps IgG Test System, 9Z9281G, ZEUS Scientific) [15], [16], [17]. The measles, mumps, and rubella tests were performed at the Laboratory of Pediatric and Respiratory Viral Diseases, US FDA, in Silver Spring, MD. JE antibody response was measured at 0 and 28 (+7) days by JE-PRNT50 with seropositive defined as ≥1:10 at the Department of Virology, Armed Forces Research Institute of Medical Sciences (AFRIMS), Bangkok, Thailand [18], [19]. Per AFRIMS standard operating procedure, the assay was conducted in LLC-MK2 cells inoculated with JE SA-14-14-2 (0423-PDK-9) obtained from the Walter Reed Army Institute of Research. Data for subjects seropositive at baseline were eliminated from the per protocol analysis of immunogenicity.

### 2.4 Primary and secondary immunogenicity outcomes

The primary outcomes were the proportion of recipients who were seropositive for measles neutralizing antibody (≥120 mIU/mL) and rubella IgG antibody (≥10 IU/mL) at 56 days post-vaccination. Secondary outcomes were the proportion of recipients who were seropositive for mumps IgG antibody (Index Value/OD Ratio ≥ 1.10) at 56 days post-vaccination and JE neutralizing antibody (≥1:10 titer) at 28 days post-vaccination with CD-JEV. Additional secondary outcomes were geometric mean concentration/titer (GMC/GMT) of measles and rubella at 56 days post-vaccination and JE antibodies at 28 days post-vaccination, respectively.

### 2.5 Safety and reactogenicity assessment

Study physicians assessed safety during a 30-minute direct observation after each vaccination, review of parent reported solicited injection site (ecchymosis, erythema, edema, induration, and pain/tenderness) and systemic adverse reactions (fever, rash, cough, runny nose, change in eating habits, diarrhea, sleepiness, irritability, unusual crying, vomiting) occurring within 14 days after each vaccination, direct observation or review of reported unsolicited AEs occurring within 28 days after each vaccination, and direct observation or review of reported serious adverse events (SAEs) occurring throughout participation in the study (until early termination or Day 112, whichever was later). All SAES were followed until resolved. To encourage accurate reporting of events, parents were called two days following each vaccination as follow-up and reminded to contact study staff if their child experienced an adverse event. An adverse event was defined as any untoward medical occurrence in a child given a study vaccine, regardless of causality. SAEs were defined as death, life-threatening event, event requiring hospitalization, event resulting in significant disability, or an event based upon medical judgement that jeopardized the health of the participant and required medical intervention.

All solicited local and systemic signs recorded from 30 min through 14 days post-vaccination were considered “related” to study vaccination. The parents used a structured reactogenicity diary card for recording solicited (pre-listed) and unsolicited reactogenicity. Any reactogenicity continuing beyond 14 days was documented as an adverse event and followed until resolution. Local and systemic signs and symptoms were documented and graded from mild to potentially life-threatening on predefined 1–4 scales based on functional assessment or magnitude of reaction [20]. All unsolicited AEs occurring within 28 days of vaccination were graded from mild to potentially life-threatening on a 1–4 scale for severity and assessed for relationship to vaccine [20].

An independent safety monitoring committee (SMC) reviewed all SAEs and evaluated such events against the known or expected safety profiles of the study vaccines and the known health of the study population. Clinical and laboratory data, clinical records, and other study-related records were made available to the SMC, as appropriate and/or available. Tables of AEs were also reviewed by the committee.

### 2.6 Statistical analysis

All immunogenicity analyses and summaries were performed on a per-protocol (PP) basis. Participants were included in the PP populations if they fulfilled eligibility criteria; were seronegative for antibody to measles, mumps, rubella, and JE viruses; received all study vaccines as assigned; had valid serology results for samples collected within assigned window periods; and received no prohibited medications 90 days before or during the study. Supportive intention-to-treat (ITT) immunogenicity analyses were also conducted on enrolled children who received at least one dose of study vaccine and had at least one post-vaccination serology result. The percentage of participants with seropositivity was calculated for each group along with its exact two-sided 95% CI obtained using Clopper-Pearson method. Seropositivity rates were compared using a non-inferiority test. Non-inferiority was achieved if the lower limit of the two-sided 95% CI for the difference in percentages of participants with seropositivity between the two groups (concurrent administration minus separate administration) at 56 days post-vaccination was >−10%. The 95% CI for the difference was calculated using the Farrington-Manning score method. The ratio of geometric mean concentrations/titers between groups was obtained by analysis of covariance with log_10_-transformed antibody concentration/titer as dependent variable and treatment group as explanatory variable adjusted for log_10_-transformed baseline antibody concentration/titer.

### 2.7 Ethical practices

The study was conducted by RITM and the study protocol and other pertinent documents were reviewed and approved by the Philippines Heart Center Institutional Review Board which served as the Philippines FDA-assigned-regulatory reviewer, as per the Philippines FDA Circular 2012-007. Likewise, this study protocol and associated amendments were reviewed by the Western Institutional Review Board (WIRB) on behalf of PATH and the RITM Institutional Ethical Review Board. Meetings were held with community leaders, local health officials, and prospective parents to inform them of the study and to solicit feedback on study procedures. Parents or guardians of 9-month-old infants were approached to give informed consent for the child’s participation in the study. Informed Consent procedures and data quality met the International Conference on Harmonisation Good Clinical Practice standards.

## 3 Results

### 3.1 Study subjects

**Fig. 1:**
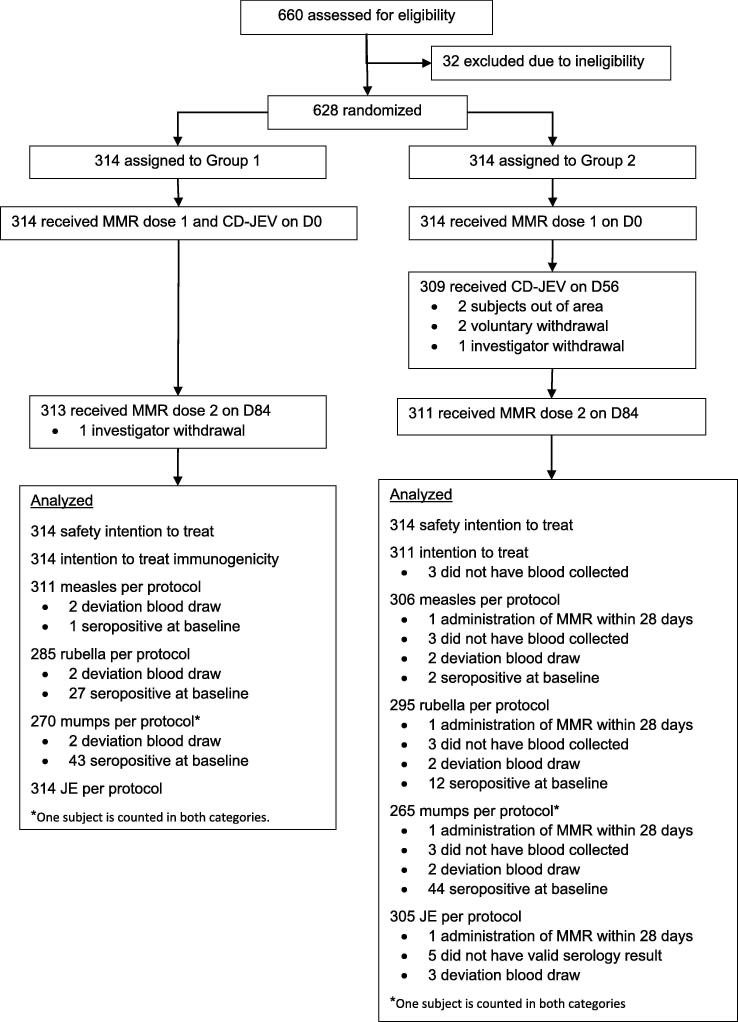
Participant disposition.

Of 660 participants screened, 628 were randomized into two groups of 314 infants. Of those randomized, 628 (100%) were vaccinated with MMR dose 1; 624 (99.4%) with MMR dose 2; 623 (99.2%) with CD-JEV; and 624 (99.4%) completed all study visits. 628 participants were included in the safety analysis, and 625 participants were evaluated in the ITT analysis for the primary outcomes. For the PP analysis, 617 (98.2%), 619 (98.6%), 580 (92.4%), and 535 (85.2%) subjects were included in the measles PP, JE PP, rubella PP, and mumps PP analyses, respectively (Fig. 1). The mean age, weight, and height of children in Groups 1 and 2 and the proportion of female enrollees in each group were not significantly different (Table 1). Medical histories regarding chronic illness, neurologic disorders, allergies, acute febrile illness within 14 days, and current medications at baseline and during the study period were similar between the two groups.

**Table 1:** Summary demographic and baseline characteristics, ITT population.

	**Statistics**	Group 1 (N = 314)*	Group 2 (N = 314)*	**Total (N = 628)**	**P-value**
Age (months)	Mean (SD)	9.3 (0.3)	9.3 (0.3)	9.3 (0.3)	0.7183
	Median	9.2	9.2	9.2	
	Min - Max	9.0–10.0†	9.0–10.0†	9.0–10.0†	

Gender at birth					
Male	n (%)	170 (54.1%)	157 (50.0%)	327 (52.1%)	0.3378
Female	n (%)	144 (45.9%)	157 (50.0%)	301 (47.9%)	
Height (cm)	Mean (SD)	70.1 (2.7)	69.9 (2.7)	70.0 (2.7)	0.3475
	Median	70.0	69.8	70.0	
	Min - Max	63.0–79.1	63.0–78.4	63.0–79.1	
Weight (kg)	Mean (SD)	8.0 (0.9)	8.0 (1.0)	8.0 (1.0)	0.6442
	Median	8.0	7.9	7.9	
	Min - Max	5.5–11.7	5.6–12.0	5.5–12.0	
Weight-for-Height	Mean Z-score	−0.499	−0.487	−0.493	0.8922

* Group 1 = coadministration; Group 2 = sequential administration.

† All participants were 9 months old and the oldest participant was one day younger than 10 months of age.

### 3.2 Immunogenicity

The proportion of children seropositive for measles and rubella antibodies in Group 1 post-immunization was noninferior to Group 2 (Table 2a), i.e., the lower bound of 95% CIs of seropositivity difference observed between Group 1 and Group 2 was not less than −10% for either antigen. Similarly, Group 1 was noninferior to Group 2 in terms of the proportion of children seropositive for mumps and JE antibodies post-immunization (Table 2a). The geometric mean concentrations of measles neutralizing antibody and rubella IgG antibody and the geometric mean titer of JE neutralizing antibody were not significantly different between Groups 1 and 2 (Table 2b).

**Table 2a:** Measles, Rubella, Mumps, and JE seropositivity after MMR and CD-JEV vaccination, PP population.

	Group 1*			Group 2*			**Group 1 - Group 2**
**Antigen**†	**N**	**Seropositivity**	**95% CI**	**N**	**Seropositivity**	**95% CI**	**% difference [95% CI]**
Measles	N = 311	305 (98.1%)	[95.8% − 99.3%]	N = 306	300 (98.0%)	[95.8% − 99.3%]	0.0% [-2.1% − 2.2%]
Rubella	N = 285	285 (100.0%)	[98.7% − 100.0%]	N = 295	294 (99.7%)	[98.1% − 100.0%]	0.3% [-0.3% − 1.0%]
Mumps	N = 270	266 (98.5%)	[96.3% − 99.6%]	N = 265	261 (98.5%)	[96.2% − 99.6%]	0.0% [-2.0% − 2.1%]
JE	N = 314	227 (72.3%)	[67.0% − 77.2%]	N = 305	208 (68.2%)	[62.6% − 73.4%]	4.1% [-3.1% − 11.3%]

* Group 1 = coadministration; Group 2 = sequential administration.

† Measles, rubella, and mumps antibodies were measured 56 days after MMR vaccination; seropositivity was defined as an antibody concentration ≥ 120 IU/mL for measles relative to NIBSC 97/648, ≥10 IU/mL for rubella, and OD Ratio ≥ 1.10 for mumps; JE antibody was measured 28 days after CD-JEV vaccination; seropositivity was defined as neutralizing antibody titer ≥ 10.

**Table 2b:** GMC/GMT after MMR and CD-JEV vaccination, PP population.

	Group 1*			Group 2*			**Group 1/Group 2**
**Antigen**†	**N**	**GMC/GMT**	**95% CI**	**N**	**GMC/GMT**	**95% CI**	Ratio*of GMC/GMT*[95% CI]
Measles (mIU/mL)	N = 311	1964.4	[1769.3–2181.0]	N = 306	1866.3	[1649.1–2112.0]	1.1 [0.9–1.2]
Rubella (IU/mL)	N = 285	230.8	[214.4–248.5]	N = 295	229.8	[210.0–251.3]	1.0 [0.9–1.1]
JE (titer)	N = 314	24.0	[20.8–27.6]	N = 305	20.3	[17.8–23.1]	1.2 [1.0–1.4]

* Group 1 = coadministration; Group 2 = sequential administration.

† Ratio was obtained after adjusting baseline antibody values. Measles and rubella antibodies were measured 56 days after MMR vaccination; JE antibody was measured 28 days after CD-JEV vaccination.

### 3.3 Safety

Concurrent immunization with CD-JEV and MMR vaccines was not associated with any unusual safety signals when compared with sequential immunization. During the study, no vaccine-associated encephalitis cases or deaths were noted in either group. There were 23 participants hospitalized for illnesses, resulting in 24 SAEs. Group 1 participants experienced 8 SAEs comprised of 7 gastroenteritis illnesses and 1 febrile convulsion that occurred 63 days after initial immunization with MMR/CD-JEV and 23 days before MMR2. Group 2 participants experienced 16 SAEs attributable to pneumonia (n = 6), gastroenteritis (n = 4), amoebiasis (n = 3), bronchitis (n = 1), urinary tract infection (n = 1), and food intolerance (n = 1). No SAEs were considered related to vaccination or determined to be potentially life-threatening.

Of 23 immediate reactions following any vaccination, 22 were mild with MMR injection site redness being the most often observed (n = 10). One child immediately had a fever classified as moderate. The proportion of subjects with immediate reactions for any vaccination in both groups were similar.

Reported solicited reactions were minimal in the 14 days following any vaccinations with reactions being similar between groups. With regard to systemic reactions within the first 14 days following receipt of CD-JEV (only), MMR (only), and CD-JEV/MMR (co-administered), there were no significant differences in the frequency of fever, rash, cough, or irritability (Table 3). Group 1 and Group 2 did not significantly differ in the frequency of solicited local reactions in the first two weeks following any vaccination or systemic reactions following the second MMR vaccine given during this study (Supplementary Appendix Tables A-E).

**Table 3:** MMR dose 1 and CD-JEV systemic reactions by maximum severity*, days 0–14, Safety population.†

		Group 1 (N = 314)‡	Group 2 (N = 314)‡	Group 2 (N = 309) ‡
Systemic Reaction	Severity	MMR/CD-JEV n (%)	MMR n (%)	CD-JEV n (%)
Fever	Any	135 (43.0%)	119 (37.9%)	87 (28.2%)
	Grade 1 or 2	90 (28.7%)	78 (24.8%)	60 (19.4%)
	Grade 3	44 (14.0%)	40 (12.7%)	26 (8.4%)
	Grade 4	1 (0.3%)	1 (0.3%)	1 (0.3%)
Rash	Any	27 (8.6%)	33 (10.5%)	13 (4.2%)
	Grade 1 or 2	26 (8.3%)	32 (10.2%)	13 (4.2%)
	Grade 3	0 (0.0%)	1 (0.3%)	0 (0.0%)
	Grade 4	1 (0.3%)	0 (0.0%)	0 (0.0%)
Cough	Any	127 (40.4%)	116 (36.9%)	118 (38.2%)
	Grade 1 or 2	123 (39.2%)	114 (36.3%)	116 (37.5%)
	Grade 3	4 (1.3%)	2 (0.6%)	2 (0.6%)
	Grade 4	0 (0.0%)	0 (0.0%)	0 (0.0%)
Diarrhea	Any	58 (18.5%)	49 (15.6%)	29 (9.4%)
	Grade 1 or 2	56 (17.8%)	47 (15.0%)	28 (9.1%)
	Grade 3	1 (0.3%)	0 (0.0%)	1 (0.3%)
	Grade 4	1 (0.3%)	2 (0.6%)	0 (0.0%)
Irritability	Any	80 (25.5%)	63 (20.1%)	36 (11.7%)
	Grade 1 or 2	77 (24.5%)	61 (19.4%)	36 (11.7%)
	Grade 3	3 (1.0%)	2 (0.6%)	0 (0.0%)
	Grade 4	0 (0.0%)	0 (0.0%)	0 (0.0%)
Vomiting	Any	29 (9.2%)	26 (8.3%)	21 (6.8%)
	Grade 1 or 2	29 (9.2%)	26 (8.3%)	21 (6.8%)
	Grade 3	0 (0.0%)	0 (0.0%)	0 (0.0%)
	Grade 4	0 (0.0%)	0 (0.0%)	0 (0.0%)

* Grade 1 = Mild; Grade 2 = Moderate; Grade 3 = Severe; Grade 4 = Potentially Life-Threatening.

† Table 3 has been abbreviated to include the more important symptoms. A more complete reporting of all symptoms is included in Table D of the Supplemental Appendix.

‡ Group 1 = coadministration; Group 2 = sequential administration.

Unsolicited AEs within 28 days of immunization were reported by: 207 (65.9%) Group 1 participants following MMR dose 1 and CD-JEV vaccination, 211 (67.2%) Group 2 participants following MMR dose 1, and 164 (53.1%) Group 2 participants following CD-JEV (Supplementary Appendix Table F). Of these reports, 97.1% (n = 201), 95.7% (n = 202), and 95.1% (n = 156) of the respective reports were due to infections and infestations. Within 28 days of MMR dose 2 vaccination, 258 (41.3%) participants reported an unsolicited adverse event of which 110 (35.1%) were Group 1 participants and 148 (47.6%) were Group 2 participants.

## 4 Discussion

Measles and rubella control are global priorities. In 2012, the World Health Assembly endorsed the Global Vaccine Action Plan to target measles and rubella elimination in multiple WHO regions by 2020 and to maximize measles vaccine coverage in the wake of numerous measles outbreaks globally [21], [22]. Introduction of new vaccines or changes in co-administered measles-containing vaccines should be studied and closely monitored to assure that there is no interference with the immune response to measles and rubella. In this study, co-administration of CD-JEV vaccine and measles-mumps-rubella vaccine in children 9- to 12-months-old did not interfere with the antibody titers against measles and rubella. As a result of this study, national immunization programs should be encouraged to co-administer MMR vaccine to 9-month-old infants with the full knowledge that CD-JEV does not interfere with the protection against measles and rubella elicited by the strains present in the MMR vaccines used in this study. Whether different MCVs made with different measles vaccine strains would result in a different outcome or whether waning JEV antibody levels can be accelerated by one measles strain as opposed to another is not known at this time. The findings in this paper support the longstanding WHO recommendations that recognize that optimal response to measles antigen may not occur as early as 9 months of age, but local disease incidence may require administration of MCV at 9 months with compensatory protection achieved by administration of MCV2 at 12 months of age [23].

Because JE vaccine may be given during the same visit when MMR is delivered, it is important to show that co-administration of MMR and CD-JEV does not decrease the immunogenicity of measles, mumps, or rubella or generate new adverse events. This study shows that these two live attenuated vaccines—MMR and CD-JEV—may be co-administered to 9-month-old children without reducing the immune response to measles, mumps, rubella, and JE antigens. Seropositivity rates for measles, mumps, and rubella were high among all groups and consistent with findings from previous studies and recommendations by WHO [24], [25], [26], [27]. GMCs for measles and rubella exceeded acceptable levels for protection as well [11], [12], [13], [27]. Likewise, concurrent vaccination with CD-JEV and MMR vaccines was not associated with any unusual safety signals when compared with sequential immunization, indicating the vaccines are safe and tolerable when given together.

In this study, the combined JE seropositivity rate for the two groups was 70.3% (95% CI: 66.5–73.9), lower than observed in two other studies also using CD-JEV from CDIBP’s Good Manufacturing Practice compliant facility where seropositivity 28 days post-vaccination ranged from 82.3% to 99.1% [14], [28]. This difference may be due to non-vaccine-related factors such as unmeasured differences between this study population and the populations in previous studies or due to a different laboratory performing the neutralizing antibody assays. Differences may be due to the cell lines used for virus propagation, cell culture media, age of complement, and the JE virus strain that is neutralized. The variability in results for JE PRNT has been well documented [29]. This variability makes comparing the results across multiple studies difficult.

The difficulty and variability of JE-PRNT assays is a limitation of this study since previous attempts to standardize the JE assay across expert laboratories have failed [29]. Although this variability limits comparison of neutralizing antibody titers when PRNTs are performed in different laboratories and may account for lower antibody responses to CD-JEV measured in this study than has been reported previously, it does not affect the validity of the group comparisons in this study [6], [7], [8], [9], [14]. Similar studies compare serology results 28 days post-vaccination for MMR and JE antigens. Although measles and mumps have typically been assayed at 28 days after immunization, the antibody levels do not drop appreciably over the second month after immunization, so we standardized the testing schedule at 56 days post-immunization for MMR to obtain the optimal antibody response detection times for rubella [11], [12], [13].

This study clearly demonstrates non-inferiority of the immune responses when administering MMR and CD-JEV concurrently in children at 9 months of age compared with sequential administration. These findings are consistent with earlier studies showing co-administration of CD-JEV with measles or measles-rubella vaccines is safe and does not lower the immunogenicity of the measles- or rubella-containing vaccine [6], [7], [8], [9]. This is important because JE vaccine and MCV are often given together in JE-endemic areas to maximize vaccine coverage against multiple diseases and because of the increasing global emphasis on measles and rubella elimination.

## CRediT authorship contribution statement

**Maria Rosario Capeding:** Conceptualization, Methodology, Validation, Investigation, Resources, Data curation, Writing - review & editing, Supervision, Project administration. **Edison Alberto:** Conceptualization, Methodology, Validation, Investigation, Resources, Data curation, Writing - review & editing, Supervision, Project administration. **Jodi Feser:** Conceptualization, Methodology, Validation, Formal analysis, Resources, Data curation, Writing - original draft, Writing - review & editing, Visualization, Supervision, Project administration. **Jessica Mooney:** Methodology, Validation, Resources, Data curation, Writing - review & editing, Visualization, Supervision, Project administration. **Yuxiao Tang:** Conceptualization, Methodology, Validation, Formal analysis, Resources, Data curation, Writing - original draft, Writing - review & editing, Visualization, Supervision, Project administration. **Susette A. Audet:** Conceptualization, Methodology, Validation, Formal analysis, Investigation, Resources, Data curation, Writing - review & editing, Supervision, Project administration. **Judy A. Beeler:** Conceptualization, Methodology, Validation, Formal analysis, Investigation, Resources, Data curation, Writing - review & editing, Supervision, Project administration. **Damon W. Ellison:** Conceptualization, Methodology, Validation, Formal analysis, Investigation, Resources, Data curation, Writing - review & editing, Supervision, Project administration. **Lei Zhang:** Conceptualization, Writing - review & editing. **G. William Letson:** Conceptualization, Methodology, Validation, Resources, Data curation, Writing - original draft, Writing - review & editing, Visualization, Supervision, Project administration. **Kathleen M. Neuzil:** Conceptualization, Methodology, Writing - review & editing, Visualization, Funding acquisition. **Anthony A. Marfin:** Conceptualization, Methodology, Validation, Resources, Data curation, Writing - original draft, Writing - review & editing, Visualization, Supervision, Project administration, Funding acquisition.

## Declaration of Competing Interest

L Zhang is employed by the manufacturer of the vaccine, Chengdu Institute of Biological Products Co., Ltd. All other authors declare no competing interests.
