# Supplementary material for: Immunogenicity and safety of concurrent or sequential administration of live, attenuated SA 14-14-2 Japanese encephalitis vaccine (CD-JEV) and measles-mumps-rubella vaccine in infants 9–12 months of age in the Philippines: A non-inferiority Phase 4 randomized clinical trial
Source: Vaccine X. 2020 Aug 14;6:100074. doi: 10.1016/j.jvacx.2020.100074 (PMC7509462; doi:10.1016/j.jvacx.2020.100074)
Supplement: Supplementary data 1 [file mmc1.docx]

Supplemental Appendix

Table A. MMR dose 1 solicited local reactions by maximum severity*, days 0-14, Safety population

|  | **Group 1 (N=314)** ^†^ | | | |  | **Group 2 (N=314)** ^†^ | | | |
| --- | --- | --- | --- | --- | --- | --- | --- | --- | --- |
|  | **None**  **n (%)** | **Grade 1**  **n (%)** | **Grade 2**  **n (%)** | **≥ Grade 3**  **n (%)** |  | **None**  **n (%)** | **Grade 1**  **n (%)** | **Grade 2**  **n (%)** | **≥ Grade 3**  **n (%)** |
| Ecchymosis | 308 (98.1%) | 6 (1.9%) | - | - |  | 304 (96.8%) | 10 (3.2%) | - | - |
| Erythema | 299 (95.2%) | 15 (4.8%) | - | - |  | 300 (95.5%) | 14 (4.5%) | - | - |
| Edema | 308 (98.1%) | 5 (1.6%) | 1 (0.3%) | - |  | 307 (97.8%) | 7 (2.2%) | - | - |
| Induration | 310 (98.7%) | 4 (1.3%) | - | - |  | 307 (97.8%) | 7 (2.2%) | - | - |
| Pain/tenderness | 296 (94.3%) | 17 (5.4%) | 1 (0.3%) | - |  | 295 (93.9%) | 17 (5.4%) | 2 (0.6%) | - |

*Grade 1 = Mild; Grade 2 = Moderate; Grade 3 = Severe; Grade 4 = Potentially Life-Threatening

† Group 1 = coadministration; Group 2 = sequential administration

Table B. MMR dose 2 solicited local reactions by maximum severity*, days 0-14, Safety population

|  | **Group 1 (N=313)**^†^ | | | |  | **Group 2 (N=311)**^†^ | | | |
| --- | --- | --- | --- | --- | --- | --- | --- | --- | --- |
|  | **None**  **n (%)** | **Grade 1**  **n (%)** | **Grade 2**  **n (%)** | **≥ Grade 3**  **n (%)** |  | **None**  **n (%)** | **Grade 1**  **n (%)** | **Grade 2**  **n (%)** | **≥ Grade 3**  **n (%)** |
| Ecchymosis | 313 (100.0%) | - | - | - |  | 306 (98.4%) | 5 (1.6%) | - | - |
| Erythema | 308 (98.4%) | 5 (1.6%) | - | - |  | 304 (97.7%) | 7 (2.3%) | - | - |
| Edema | 313 (100.0%) | - | - | - |  | 307 (98.7%) | 4 (1.3%) | - | - |
| Induration | 313 (100.0%) | - | - | - |  | 308 (99.0%) | 3 (1.0%) | - | - |
| Pain/tenderness | 305 (97.4%) | 8 (2.6%) | - | - |  | 306 (98.4%) | 4 (1.3%) | 1 (0.3%) | - |

*Grade 1 = Mild; Grade 2 = Moderate; Grade 3 = Severe; Grade 4 = Potentially Life-Threatening

† Group 1 = coadministration; Group 2 = sequential administration

Table C. CD-JEV solicited local reactions by maximum severity*, days 0-14, Safety population

|  | **Group 1 (N=314)**^†^ | | | |  | **Group 2 (N=309)**^†^ | | | |
| --- | --- | --- | --- | --- | --- | --- | --- | --- | --- |
|  | **None**  **N (%)** | **Grade 1**  **N (%)** | **Grade 2**  **N (%)** | **≥ Grade 3**  **N (%)** |  | **None**  **N (%)** | **Grade 1**  **N (%)** | **Grade 2**  **N (%)** | **≥Grade 3**  **N (%)** |
| Ecchymosis | 308 (98.1) | 6 (1.9) | - | - |  | 306 (99.0) | 3 (1.0) | - | - |
| Erythema | 301 (95.9) | 12 (3.8) | 1 (0.3) | - |  | 301 (97.4) | 8 (2.6) | - | - |
| Edema | 311 (99.0) | 3 (1.0) | - | - |  | 304 (98.4) | 5 (1.6) | - | - |
| Induration | 307 (97.8) | 6 (1.9) | 1 (0.3) | - |  | 305 (98.7) | 4 (1.3) | - | - |
| Pain/tenderness | 296 (94.3) | 17 (5.4) | 1 (0.3) | - |  | 303 (98.1) | 5 (1.6) | 1 (0.3) | - |

*Grade 1 = Mild; Grade 2 = Moderate; Grade 3 = Severe; Grade 4 = Potentially Life-Threatening

† Group 1 = coadministration; Group 2 = sequential administration

Table D. MMR dose 1 and CD-JEV systemic reactions by maximum severity*, days 0-14, Safety population

|  |  | **Group 1 (N=314)**^†^ |  | **Group 2 (N=314)**^†^ | **Group 2 (N=309)**^†^ |
| --- | --- | --- | --- | --- | --- |
| **Systemic Reaction** | **Severity** | **MMR/CD-JEV**  **n (%)** |  | **MMR**  **n (%)** | **CD-JEV**  **n (%)** |
| Fever | Any | 135 (43.0%) |  | 119 (37.9%) | 87 (28.2%) |
|  | Grade 1 or 2 | 90 (28.7%) |  | 78 (24.8%) | 60 (19.4%) |
|  | Grade 3 | 44 (14.0%) |  | 40 (12.7%) | 26 (8.4%) |
|  | Grade 4 | 1 (0.3%) |  | 1 (0.3%) | 1 (0.3%) |
| Rash | Any | 27 (8.6%) |  | 33 (10.5%) | 13 (4.2%) |
|  | Grade 1 or 2 | 26 (8.3%) |  | 32 (10.2%) | 13 (4.2%) |
|  | Grade 3 | 0 (0.0%) |  | 1 (0.3%) | 0 (0.0%) |
|  | Grade 4 | 1 (0.3%) |  | 0 (0.0%) | 0 (0.0%) |
| Cough | Any | 127 (40.4%) |  | 116 (36.9%) | 118 (38.2%) |
|  | Grade 1 or 2 | 123 (39.2%) |  | 114 (36.3%) | 116 (37.5%) |
|  | Grade 3 | 4 (1.3%) |  | 2 (0.6%) | 2 (0.6%) |
|  | Grade 4 | 0 (0.0%) |  | 0 (0.0%) | 0 (0.0%) |
| Runny nose | Any | 167 (53.2%) |  | 163 (51.9%) | 143 (46.3%) |
|  | Grade 1 or 2 | 165 (52.5%) |  | 161 (51.3%) | 142 (46.0%) |
|  | Grade 3 | 2 (0.6%) |  | 2 (0.6%) | 1 (0.3%) |
|  | Grade 4 | 0 (0.0%) |  | 0 (0.0%) | 0 (0.0%) |
| Change in eating habits | Any | 49 (15.6%) |  | 32 (10.2%) | 27 (8.7%) |
|  | Grade 1 or 2 | 49 (15.6%) |  | 31 (9.9%) | 27 (8.7%) |
|  | Grade 3 | 0 (0.0%) |  | 1 (0.3%) | 0 (0.0%) |
|  | Grade 4 | 0 (0.0%) |  | 0 (0.0%) | 0 (0.0%) |
| Diarrhea | Any | 58 (18.5%) |  | 49 (15.6%) | 29 (9.4%) |
|  | Grade 1 or 2 | 56 (17.8%) |  | 47 (15.0%) | 28 (9.1%) |
|  | Grade 3 | 1 (0.3%) |  | 0 (0.0%) | 1 (0.3%) |
|  | Grade 4 | 1 (0.3%) |  | 2 (0.6%) | 0 (0.0%) |
| Sleepiness | Any | 43 (13.7%) |  | 32 (10.2%) | 12 (3.9%) |
|  | Grade 1 or 2 | 43 (13.7%) |  | 32 (10.2%) | 12 (3.9%) |
|  | Grade 3 or 4 | 0 (0.0%) |  | 0 (0.0%) | 0 (0.0%) |
| Irritability | Any | 80 (25.5%) |  | 63 (20.1%) | 36 (11.7%) |
|  | Grade 1 or 2 | 77 (24.5%) |  | 61 (19.4%) | 36 (11.7%) |
|  | Grade 3 | 3 (1.0%) |  | 2 (0.6%) | 0 (0.0%) |
|  | Grade 4 | 0 (0.0%) |  | 0 (0.0%) | 0 (0.0%) |
| Unusual crying | Any | 54 (17.2%) |  | 29 (9.2%) | 20 (6.5%) |
|  | Grade 1 or 2 | 53 (16.9%) |  | 28 (8.9%) | 20 (6.5%) |
|  | Grade 3 | 1 (0.3%) |  | 1 (0.3%) | 0 (0.0%) |
|  | Grade 4 | 0 (0.0%) |  | 0 (0.0%) | 0 (0.0%) |
| Vomiting | Any | 29 (9.2%) |  | 26 (8.3%) | 21 (6.8%) |
|  | Grade 1 or 2 | 29 (9.2%) |  | 26 (8.3%) | 21 (6.8%) |
|  | Grade 3 or 4 | 0 (0.0%) |  | 0 (0.0%) | 0 (0.0%) |

*Grade 1 = Mild; Grade 2 = Moderate; Grade 3 = Severe; Grade 4 = Potentially Life-Threatening

† Group 1 = coadministration; Group 2 = sequential administration

Table E. MMR dose 2 systemic reactions by maximum severity*, days 0-14, Safety population

|  |  | **Group 1 (N=313)**^†^ |  | **Group 2 (N=311)** ^†^ |  | **Total (N=624)** |
| --- | --- | --- | --- | --- | --- | --- |
| **Systemic Reaction** | **Severity** | **MMR**  **n (%)** |  | **MMR**  **n (%)** |  | **MMR**  **n (%)** |
| Fever | Any | 79 (25.2%) |  | 84 (27.0%) |  | 163 (26.1%) |
|  | Grade 1 or 2 | 54 (17.3%) |  | 51 (16.4%) |  | 105 (16.8%) |
|  | Grade 3 | 25 (8.0%) |  | 32 (10.3%) |  | 57 (9.1%) |
|  | Grade 4 | 0 (0.0%) |  | 1 (0.3%) |  | 1 (0.2%) |
| Rash | Any | 19 (6.1%) |  | 18 (5.8%) |  | 37 (5.9%) |
|  | Grade 1 or 2 | 18 (5.8%) |  | 18 (5.8%) |  | 36 (5.8%) |
|  | Grade 3 | 0 (0.0%) |  | 0 (0.0%) |  | 0 (0.0%) |
|  | Grade 4 | 1 (0.3%) |  | 0 (0.0%) |  | 1 (0.2%) |
| Cough | Any | 111 (35.5%) |  | 103 (33.1%) |  | 214 (34.3%) |
|  | Grade 1 or 2 | 111 (35.5%) |  | 103 (33.1%) |  | 214 (34.3%) |
|  | Grade 3 or 4 | 0 (0.0%) |  | 0 (0.0%) |  | 0 (0.0%) |
| Runny nose | Any | 133 (42.5%) |  | 135 (43.4%) |  | 268 (42.9%) |
|  | Grade 1 or 2 | 131 (41.9%) |  | 135 (43.4%) |  | 266 (42.6%) |
|  | Grade 3 | 2 (0.6%) |  | 0 (0.0%) |  | 2 (0.3%) |
|  | Grade 4 | 0 (0.0%) |  | 0 (0.0%) |  | 0 (0.0%) |
| Change in eating habits | Any | 36 (11.5%) |  | 27 (8.7%) |  | 63 (10.1%) |
|  | Grade 1 or 2 | 35 (11.2%) |  | 26 (8.4%) |  | 61 (9.8%) |
|  | Grade 3 | 1 (0.3%) |  | 1 (0.3%) |  | 2 (0.3%) |
|  | Grade 4 | 0 (0.0%) |  | 0 (0.0%) |  | 0 (0.0%) |
| Diarrhea | Any | 34 (10.9%) |  | 36 (11.6%) |  | 70 (11.2%) |
|  | Grade 1 or 2 | 31 (9.9%) |  | 34 (10.9%) |  | 65 (10.4%) |
|  | Grade 3 | 2 (0.6%) |  | 1 (0.3%) |  | 3 (0.5%) |
|  | Grade 4 | 1 (0.3%) |  | 1 (0.3%) |  | 2 (0.3%) |
| Sleepiness | Any | 15 (4.8%) |  | 16 (5.1%) |  | 31 (5.0%) |
|  | Grade 1 or 2 | 15 (4.8%) |  | 16 (5.1%) |  | 31 (5.0%) |
|  | Grade 3 or 4 | 0 (0.0%) |  | 0 (0.0%) |  | 0 (0.0%) |
| Irritability | Any | 46 (14.7%) |  | 33 (10.6%) |  | 79 (12.7%) |
|  | Grade 1 or 2 | 44 (14.1%) |  | 32 (10.3%) |  | 76 (12.2%) |
|  | Grade 3 | 2 (0.6%) |  | 1 (0.3%) |  | 3 (0.5%) |
|  | Grade 4 | 0 (0.0%) |  | 0 (0.0%) |  | 0 (0.0%) |
| Unusual crying | Any | 29 (9.3%) |  | 20 (6.4%) |  | 49 (7.9%) |
|  | Grade 1 or 2 | 29 (9.3%) |  | 19 (6.1%) |  | 48 (7.7%) |
|  | Grade 3 | 0 (0.0%) |  | 1 (0.3%) |  | 1 (0.2%) |
|  | Grade 4 | 0 (0.0%) |  | 0 (0.0%) |  | 0 (0.0%) |
| Vomiting | Any | 22 (7.0%) |  | 31 (10.0%) |  | 53 (8.5%) |
|  | Grade 1 or 2 | 19 (6.1%) |  | 29 (9.3%) |  | 48 (7.7%) |
|  | Grade 3 | 2 (0.6%) |  | 1 (0.3%) |  | 3 (0.5%) |
|  | Grade 4 | 1 (0.3%) |  | 1 (0.3%) |  | 2 (0.3%) |

*Grade 1 = Mild; Grade 2 = Moderate; Grade 3 = Severe; Grade 4 = Potentially Life-Threatening

† Group 1 = coadministration; Group 2 = sequential administration

Table F Summary of adverse events reported within 28 days of MMR dose 1 and CD-JEV, Safety Population

|  | **Group 1 (N=314)*** |  | **Group 2 (N=314)*** | **Group 2 (N=309)*** |
| --- | --- | --- | --- | --- |
| **System Organ Class** | **MMR-CD/JEV** |  | **MMR** | **CD-JEV** |
| All Systems | 207 (65.9%) |  | 211 (67.2%) | 164 (53.1%) |
| Blood and lymphatic system disorders | 0 (0.0%) |  | 1 (0.3%) | 0 (0.0%) |
| Eye disorders | 1 (0.3%) |  | 0 (0.0%) | 0 (0.0%) |
| Gastrointestinal disorders | 6 (1.9%) |  | 4 (1.3%) | 4 (1.3%) |
| General disorders | 5 (1.6%) |  | 2 (0.6%) | 5 (1.6%) |
| Immune system disorders | 4 (1.3%) |  | 3 (1.0%) | 1 (0.3%) |
| Infections and infestations | 201 (64.0%) |  | 202 (64.3%) | 156 (50.5%) |
| Injury, poisoning and procedural complications | 7 (2.2%) |  | 2 (0.6%) | 2 (0.6%) |
| Metabolism and nutrition disorders | 0 (0.0%) |  | 1 (0.3%) | 1 (0.3%) |
| Nervous system disorders | 0 (0.0%) |  | 0 (0.0%) | 0 (0.0%) |
| Psychiatric disorders | 1 (0.3%) |  | 0 (0.0%) | 0 (0.0%) |
| Respiratory, thoracic and mediastinal disorders | 2 (0.6%) |  | 3 (1.0%) | 5 (1.6%) |
| Skin and subcutaneous tissue disorders | 3 (1.0%) |  | 8 (2.5%) | 6 (1.9%) |

* Group 1 = coadministration; Group 2 = sequential administration
